# A novel nomogram based on clinical blood indicators for prognosis prediction in curatively resected esophagogastric junction adenocarcinoma patients

**DOI:** 10.7150/jca.83588

**Published:** 2023-05-21

**Authors:** Can-Tong Liu, Xin-Yi Huang, Bin-Liang Huang, Chao-Qun Hong, Hai-Peng Guo, Hong Guo, Ling-Yu Chu, Yi-Wei Lin, Yi-Wei Xu, Yu-Hui Peng, Fang-Cai Wu

**Affiliations:** 1 Department of Clinical Laboratory Medicine, Cancer Hospital of Shantou University Medical College, Shantou 515041, Guangdong, China.; 2 Esophageal Cancer Prevention and Control Research Center, Cancer Hospital of Shantou University Medical College, Shantou 515041, Guangdong, China.; 3 Guangdong Esophageal Cancer Research Institute, Guangzhou 510060, Guangdong, China.; 4 Department of Gastrointestinal Endoscopy, First Affiliated Hospital of Shantou University Medical College, Shantou 515041, Guangdong, China.; 5 Department of Head and Neck Surgery, Cancer Hospital of Shantou University Medical College, Shantou 515041, Guangdong, China.; 6 Department of Radiation Oncology, Cancer Hospital of Shantou University Medical College, Shantou 515041, Guangdong, China.

**Keywords:** Esophagogastric junction adenocarcinoma, Clinical laboratory blood indicators, Nomogram, Prognosis, Prediction

## Abstract

**Background:** The incidence of esophagogastric junction adenocarcinoma (EJA) patients was increasing but their prognoses were poor. Blood-based predictive biomarkers were associated with prognosis. This study was to build a nomogram based on preoperative clinical laboratory blood biomarkers for predicting prognosis in curatively resected EJA.

**Methods:** Curatively resected EJA patients, recruited between 2003 and 2017 in the Cancer Hospital of Shantou University Medical College, were divided chronologically into the training (n=465) and validation groups (n=289). Fifty markers, involving sociodemographic characteristics and preoperative clinical laboratory blood indicators, were screened for nomogram construction. Independent predictive factors were selected using Cox regression analysis and then were combined to build a nomogram to predict overall survival (OS).

**Results:** Composed of 12 factors, including age, body mass index, platelets, aspartate aminotransferase-to-alanine transaminase ratio, alkaline phosphatase, albumin, uric acid, IgA, IgG, complement C3, complement factor B and systemic immune-inflammation index, we constructed a novel nomogram for OS prediction. In the training group, when combined with TNM system, it acquired a C-index of 0.71, better than using TNM system only (C-index: 0.62, p < 0.001). When applied in the validation group, the combined C-index was 0.70, also better than using TNM system (C-index: 0.62, p < 0.001). Calibration curves exhibited that the nomogram-predicted probabilities of 5-year OS were both in consistency with the actual 5-year OS in both groups. Kaplan-Meier analysis exhibited that patients with higher nomogram scores contained poorer 5-year OS than those with lower scores (p < 0.0001).

**Conclusions:** In conclusion, the novel nomogram built based on preoperative blood indicators might be the potential prognosis prediction model of curatively resected EJA.

## Background

According to the latest cancer statistics, esophageal and gastric cancers rank sixth and fourth respectively in worldwide cancer mortality 1. First classified by Siewert in 1998, esophagogastric junction adenocarcinoma (EJA) was seen as an independent malignant disease with tumor center located within the 10-centimeter area between the esophagus and stomach 2. Recently, the incidence of esophageal and gastric cancers has steadily declined with more attention paid to the treatment of *Helicobacter pylori* and the change of life habits, while the incidence of EJA has increased on the contrary around the world during recent decades 3. Along with it, the poor prognosis of EJA was posing threat to the global disease burden. Compared to the 5-year overall survival (OS) rate of nearly 50% in advanced gastroesophageal cancer patients after receiving surgery, the 5-year OS rate of advanced EJA patients after surgery is worse, being not more than 30% 4.

Precise pretreatment prognostic prediction would bring benefit to EJA patients, especially those who were able and willing to undergo surgery. Recent publications have identified some novel independent prognostic predictive factors based on preoperative blood indicators in EJA patients, including C-reactive protein-to-albumin ratio (CRP/ALB), systemic immune-inflammation index (SII) and prognostic nutritional index (PNI) 5-7. For example, Kudou et al. identified that PNI and CRP/ALB were strongly associated with underprivileged prognosis in EJA or upper gastric cancer patients who underwent surgery 5, 7. However, the obvious shortcomings of these studies included the lack of biomarker combinations and a limited number of EJA samples. Therefore, it is compulsory to find a potential objective way based on clinical laboratory blood-related indicators to predict the prognosis of EJA patients.

Recently, nomogram has been explored as a method for predicting cancer outcomes 8. Increasing numbers of publications showed that some newly-constructed nomograms could provide a more precise prognosis prediction than the traditional cancer TNM system 9, 10. Wei et al. have constructed a novel nomogram in EJA patients who underwent curative resection containing TNM staging system, body mass index (BMI) and neutrophil-to-lymphocyte ratio (NLR), which showed better discrimination and calibration ability compared with TNM system 11. However, validation was lacking in their study. Therefore, we would try to construct and validate another novel nomogram for individual prognosis prediction in curatively resected EJA patients.

## Methods

### Participants enrollment

In this retrospective study, from 2003 to 2017, EJA patients, who received curative surgical resection at their first time of admission, were enrolled at the Cancer Hospital of Shantou University Medical College. Patients enrolled between 2003 and 2010 were assigned to the training group, and other patients diagnosed between 2011 and 2017 were enrolled in the validation group. Detailed inclusion criteria were as follows: patients (1) aged 40 - 80 years old; (2) receiving curatively surgical resection; (3) diagnosis of EJA by pathology after surgery; (4) with complete sociodemographic data, preoperative clinical laboratory blood data, and follow-up data. Detailed exclusion criteria were as follows: patients (1) with a previous history of any cancers or cancer-related treatments; (2) suffering from autoimmune diseases, renal failure, or infectious diseases; (3) with distant metastasis. The ethics committee of the Cancer Hospital of Shantou University Medical College passed the ethical approval (ethics number: 2023006). The research was conducted according to the Declaration of Helsinki.

### Data collection

The data for analysis were comprised of relevant information on pathological data (tumor size, invasive depth, lymph node status, and Siewert's classification), sociodemographic data, and preoperative clinical laboratory blood data within one week (complete blood cell count and biochemical parameters). Among them, the sociodemographic data included age, gender, body mass index (BMI), family tumor history, smoking history, and alcohol consumption history.

The complete blood cell count included WBC, the classification of WBC, RBC, RBC-related indicators, and platelets (PLT). The classification of WBC contained the percentage of lymphocytes (LY%), absolute lymphocyte count (LY), the percentage of monocytes (MO%), absolute monocyte count (MO), the percentage of neutrophils (NE%), and absolute neutrophil count (NE). RBC-related indicators included hemoglobin (HGB), hematocrit (HCT), mean corpuscular volume (MCV), mean corpuscular hemoglobin (MCH), and mean corpuscular hemoglobin concentration (MCHC).

In the collected biochemical biomarkers, creatine kinase (CK), hydroxybutyrate dehydrogenase (HBDH), and lactate dehydrogenase (LDH) were the representative of heart function. Ten liver function-related indicators involved alanine transaminase (ALT), aspartate aminotransferase (AST), gamma-glutamyl transpeptidase (GGT), alkaline phosphatase (ALP), total protein (TP), globin (GLB), albumin (ALB), total bilirubin (TBIL), indirect bilirubin (IBIL), and direct bilirubin (DBIL). Creatinine (SCr), glucose (FBG), and uric acid (UA) were also enrolled as the renal function-related indicators. Immune-related parameters, containing IgG, IgM, IgA, complement C3 (C3), complement factor B (CFB), and C-reactive protein (CRP), were also considered.

Some parameters reported in the previous review were also listed in this study, including NLR, lymphocyte-to-monocyte ratio (LMR), platelet-to-lymphocyte ratio (PLR), albumin-to-globin ratio (AGR), CRP/ALB, SII and PNI 12. In short, SII was calculated using the formula (SII = NE × LY/PLT), while PNI was calculated by serum ALB (g/l) plus five times LY (×10^9^/l).

The TNM stage was defined based on the eighth edition of the American Joint Committee on Cancer Staging Manual 13. In brief, if the tumor center of EJA was less than 2 cm from the esophagogastric junction, it was staged according to the esophageal cancer staging system. Those more than 2 centimeters away from the esophagogastric junction were staged using the gastric cancer staging system. Siewert's types were defined using the surgical record combined with the pathology reports by the clinicians. All enrolled patients received follow-up. OS was defined from the date of receiving surgery to death. Data were censored for patients who were still alive in December 2019.

### Survival prediction nomogram construction

When the clinical laboratory indicators were applied as prognostic indicators, the normal and abnormal classification used for diagnostic purpose appeared not to suit for distinguishing different survival risks, of which the different cutoff values were redefined in many published studies 14, 15. Therefore, in the present study, we altered the continuous variables into dichotomous variables using the new cutoff values which were calculated from the *survivalROC* package (https://cran.r-project.org/package=survivalROC) in the R project by maximizing the Youden index (sensitivity plus specificity minus one). Then univariate and multivariate Cox regression analysis were applied to screen out the potential independent prognosis predictive factors. Combining the independent predictive factors, a nomogram for predicting the OS of EJA patients was built.

### Assessment of the nomogram

We used Harrell's consistent index (C-index) to assess the discrimination performance of the novel constructed nomogram. Bootstrapping method was applied to evaluate the accuracy of the nomogram as internal validation. Calibration curves were plotted by calculating the margin effect and were to evaluate the average prediction probability of the nomogram. Net reclassification improvement (NRI) and integrated discrimination improvement (IDI) were used to calculate the precise improvement in prognosis prediction of the nomogram compared with the traditional TNM staging system. After calculating the nomogram scores of EJA patients based on the nomogram, we further utilized *survivalROC* package to identify the best cutoff value for distinguishing different prognostic risks of EJA patients.

### Statistical analysis

All statistical analyses were performed using the following softwares: IBM SPSS Statistics 24.0, Microsoft Excel, and R version 3.6.1 (http://www.R-project.org/). The Chi-square test (for categorical valuables) and the Students' t test (for continuous valuables) were used to compare the distribution difference between training and validation groups. Nomogram and calibration curves were plotted using the *rms* package (https://cran.r-project.org/package=rms), while NRI and IDI were performed using the *survIDINRI* package (https://cran.r-project.org/package=survIDINRI). Using Kaplan-Meier survival analysis, survival curves were plotted and compared using log-rank test through the *survminer* packages (https://cran.r-project.org/package=survminer). A p lower than 0.05 (two-tailed) was seen as statistical significance in this study.

## Results

### Patient enrollment

**Figure 1:**
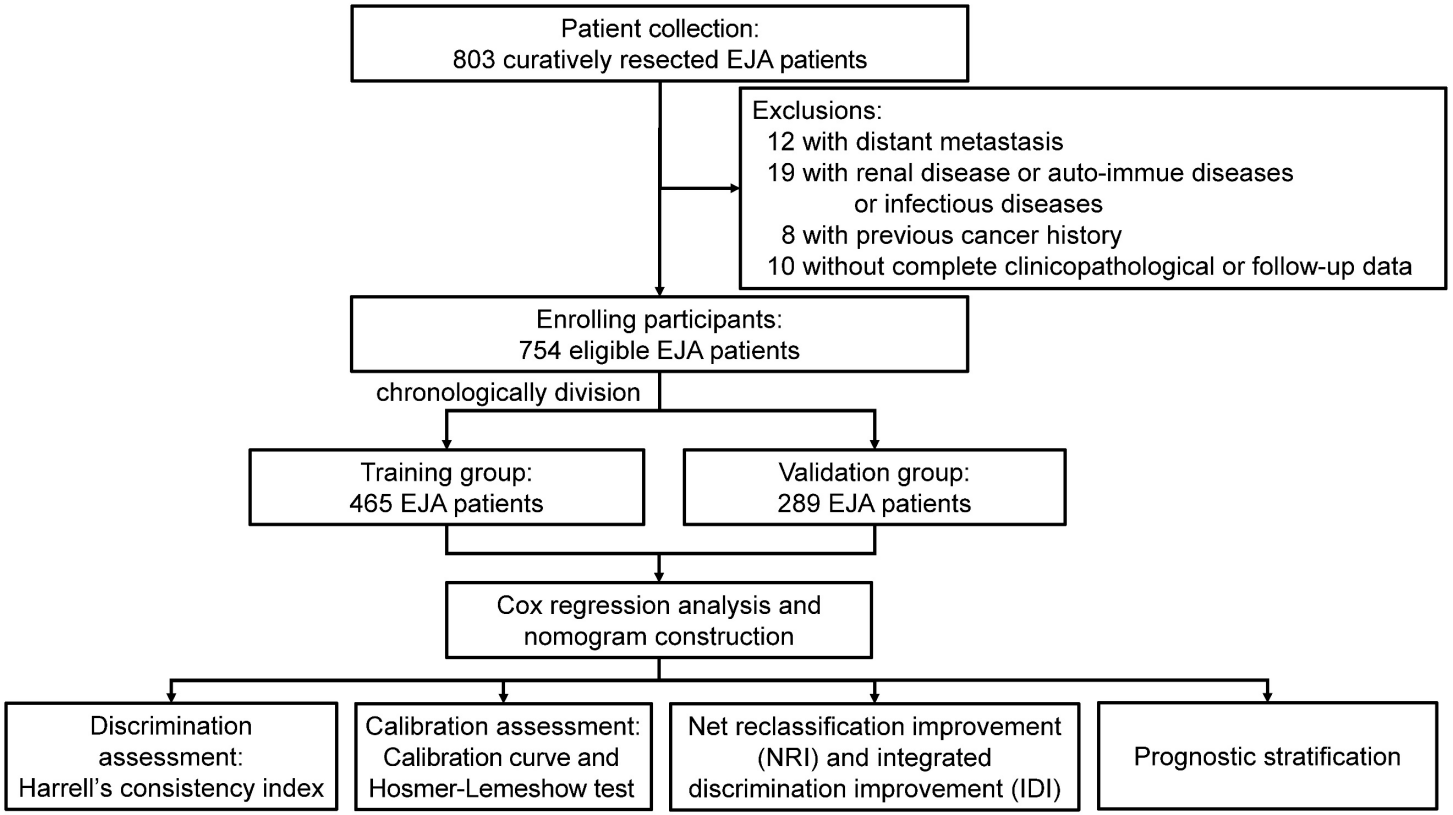
Recruitment process of EJA patients. EJA: esophagogastric junction adenocarcinoma.

After excluding the non-eligible patients, there were 754 EJA patients enrolled in our study (Figure 1). Among these, 465 patients from 2003 to 2010 were placed in the training group, and the validation group was composed of the remaining 289 patients from 2011 to 2017. As shown in Table 1, except age (*p* < 0.001), there were no significant distribution differences between both groups on gender, BMI, tumor family, smoking history, drinking history, TNM stage, and Siewert's classification (all *p* > 0.05).

### Nomogram construction for survival prediction

**Figure 2:**
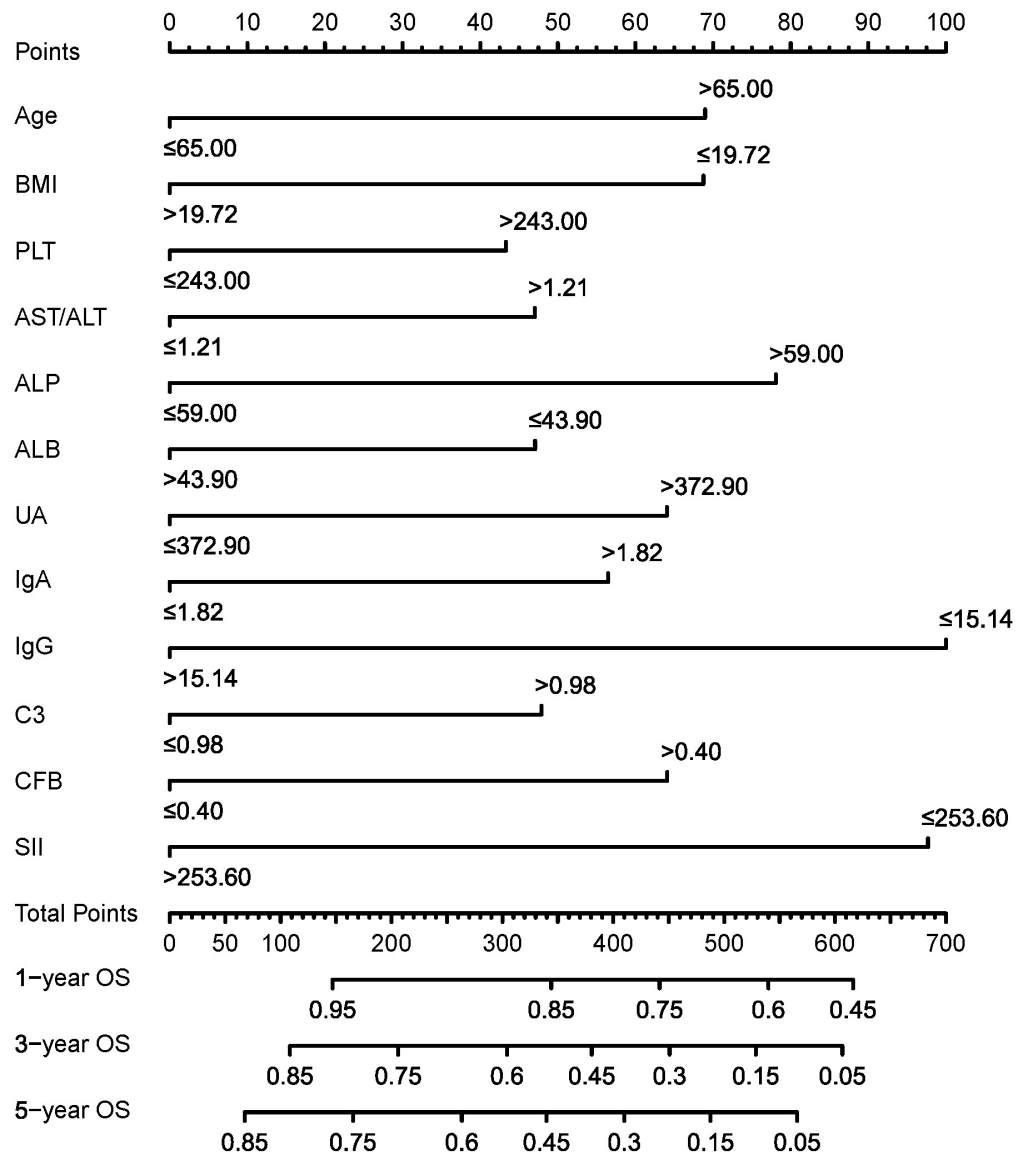
Nomogram for predicting OS of EJA patients.

As shown in Table 1, the median survival month of EJA patients was 39.2 ± 5.9 months with the 5-year OS of 44.3% in the training group, which was similar to those in the validation group (38.0 ± 6.6 months and 5-year OS of 43.7%). Then, we used univariate Cox regression analysis to select seventeen markers with *p*-values of less than 0.05 for stepwise backward multivariate Cox analysis (Supplementary Table S1). Finally, twelve independent prognostic predictive indicators were used to construct the survival prediction nomogram for EJA patients (Figure 2). These twelve indicators included age, BMI, PLT, AST/ALT, ALP, ALB, UA, IgA, IgG, C3, CFB, and SII.

### Discrimination evaluation of nomogram

After constructing the nomogram, we used C-index to evaluate its discrimination. The C-index of the constructed nomogram was 0.65 (95% CI: 0.62 ~ 0.68) in the training group, higher than using the TNM system (0.62; 95% CI: 0.59 ~ 0.65; *p* = 0.044) (Table 2). After combining the nomogram and TNM system, the C-index in prognostic prediction rose to 0.71 (95% CI: 0.68 ~ 0.74), which was also higher than using the TNM staging system alone (*p* < 0.001). In the validation group, the C-index of the nomogram/TNM combination was 0.70 (95% CI: 0.66 ~ 0.74), still higher than the TNM system alone (*p* < 0.001).

### Calibration and improvement assessment of nomogram

**Figure 3:**
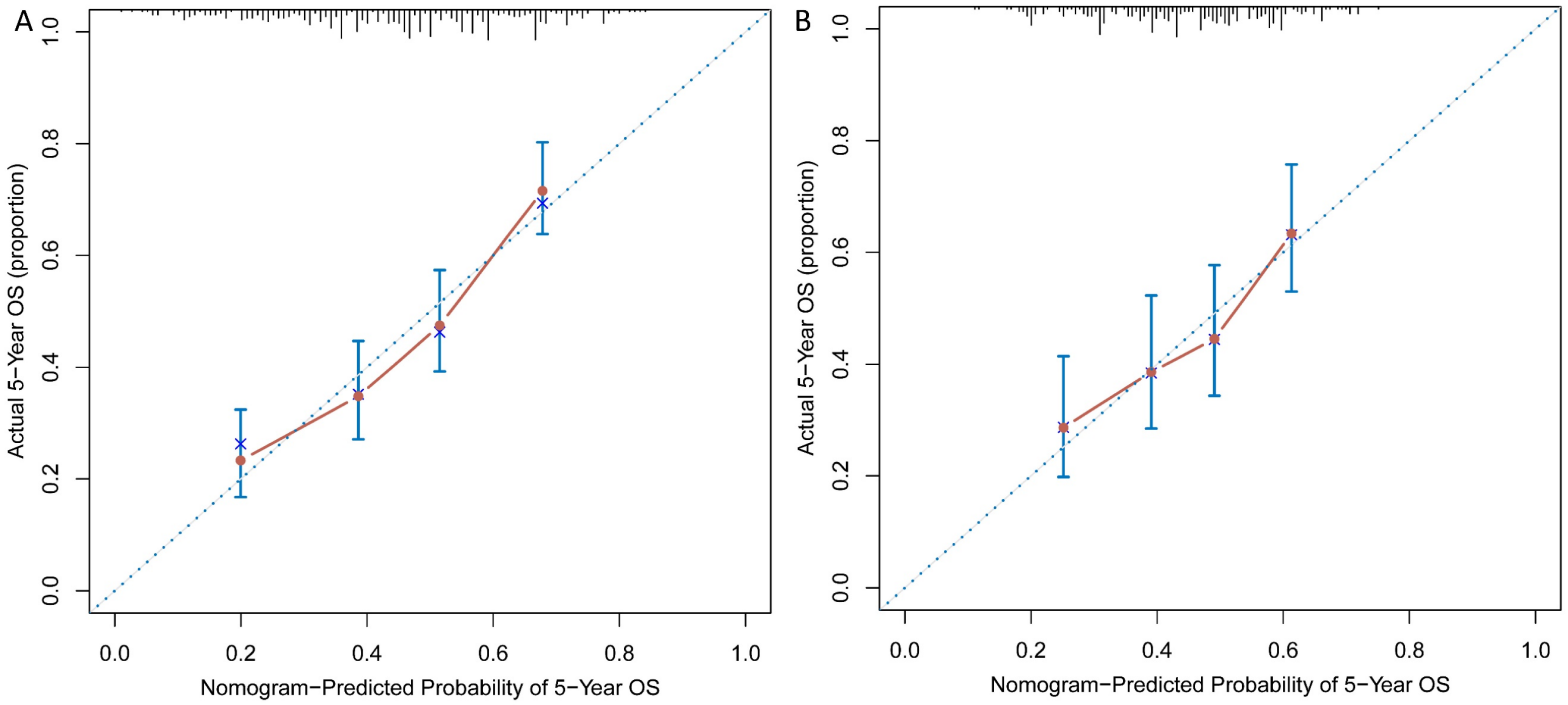
Calibration curves were plotted for the training (A) and validation (B) groups.

**Figure 4:**
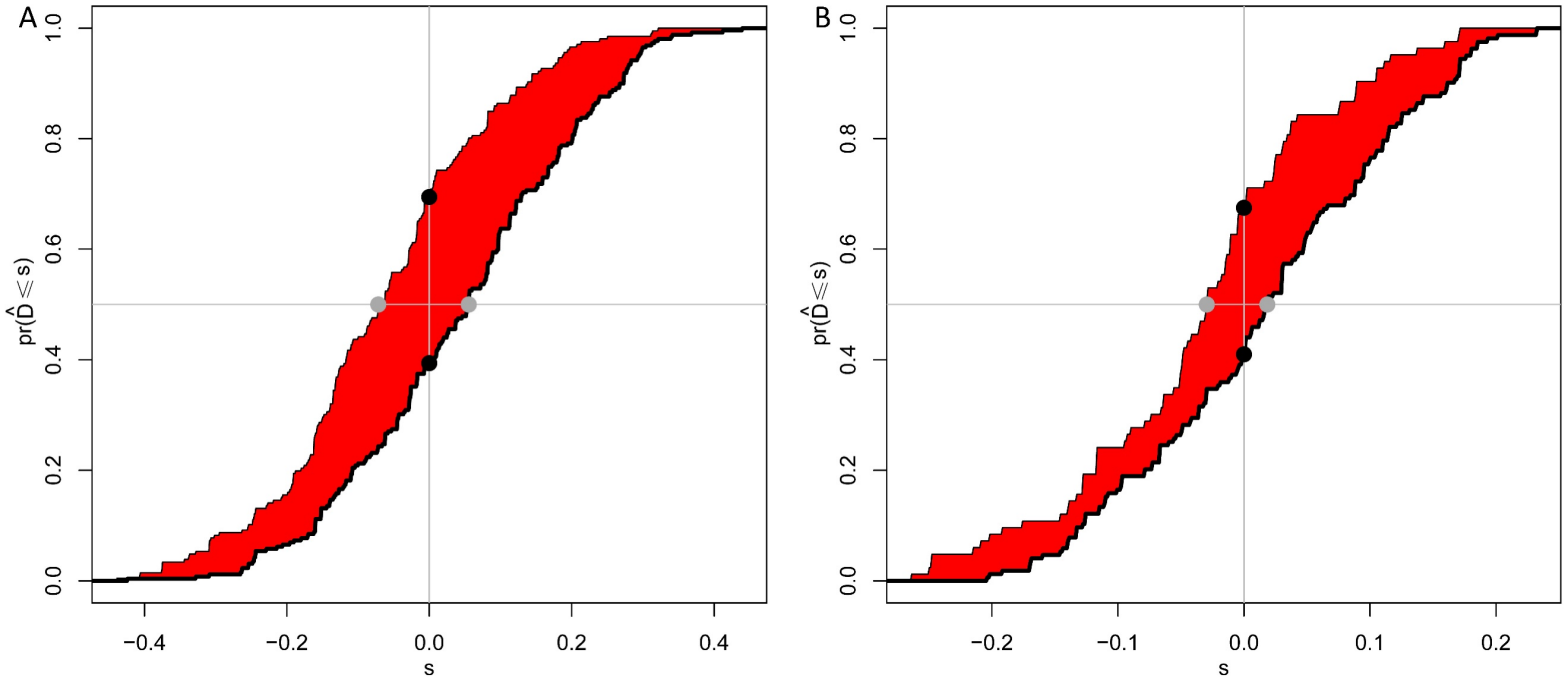
IDI and NRI of the combination of the OS nomogram and TNM system in predicting 5-year OS of EJA patients for the training (A) and validation (B) groups. The red area is Integrated discrimination improvement (IDI), the added predicted value. The distance between the two black dots represents net reclassification improvement (NRI).

As shown in Figure 3A and 3B, calibration curves exhibited that the nomogram-predicted probabilities of 5-year OS were both in consistency with the actual 5-year OS in both groups. Then, NRI and IDI were further applied to evaluate the precise prognosis predictive improvement of the TNM/nomogram combination compared with the TNM system alone. Figure 4A showed that the accuracy of the nomogram/TNM combination was better in predicting 5-year OS in the training group (NRI = 0.30, 95% CI: 0.20 ~ 0.39, *p* < 0.001; IDI = 0.11, 95% CI: 0.07 ~ 0.16, *p* < 0.001). The similar results could be found in the validation group (Figure 4B; NRI = 0.27, 95% CI: 0.09 ~ 0.40, *p* = 0.008; IDI = 0.05, 95% CI: 0.02 ~ 0.11, *p* = 0.004).

### Prognostic stratification according to nomogram score

**Figure 5:**
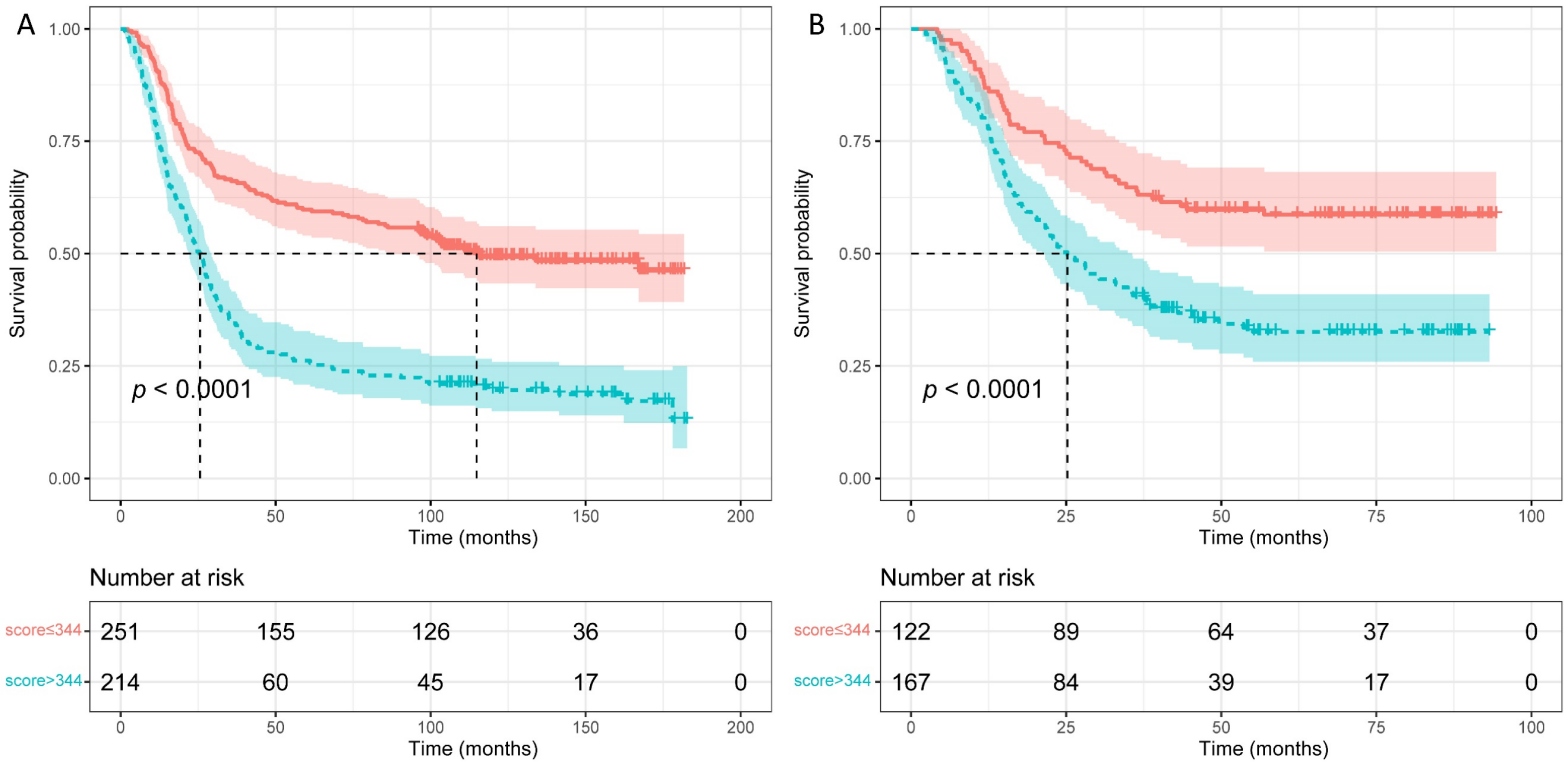
Survival curves were plotted using Kaplan-Meier survival analysis and compared by the log-rank test based on the training (A) and validation (B) groups.

To judge if the novel constructed nomogram could stratify EJA patients based on the different prognostic risks, we calculated the nomogram scores of all EJA patients. Then we used the *survivalROC* package to obtain the best nomogram score cut-off value (a score of 344) for distinguishing high- and low-risk patients. From Figure 5, the nomogram could separate the high-risk patients, and patients with high scores (> 344 scores) had poorer 5-year OS than those with low scores (in the training group: 59.6% versus 26.2%; in the validation group: 58.6% versus 38.6%; *p* < 0.001).

## Discussion

In the present study, composed of twelve factors, we built a novel nomogram based on the preoperative clinical laboratory test indicators for OS prediction in curatively resected EJA patients. In twelve independent prognostic predictive indicators, BMI, ALB and UA were all nutrition-related indicators, while seven parameters, including PLT, ALP, IgA, IgG, C3, CFB and SII, were all associated with inflammation and immune system. In the training and validation group, when combined with the TNM system, the nomogram acquired C-indexes of 0.71 and 0.70, respectively, better than using the TNM system only. Calibration curves exhibited that the nomogram-predicted probabilities of 5-year OS were both in consistency with the actual 5-year OS in both groups. Kaplan-Meier analysis exhibited that patients with higher nomogram scores contained poorer 5-year OS than those with lower scores (*p* < 0.0001).

In 1992, it has been proposed that angiogenesis was associated with metastasis and occurrence of breast cancer. As a pointer of angiogenesis, platelet endothelial cell adhesion factor (PECAF) could be used to estimate the poor prognosis of breast cancer patients 16. PECAF could express on the external surface of platelets, neutrophils, and endothelial cells. In the previous studies of colon and lung cancers, platelet counts were found to be related to lymph node status 17, 18. Platelets can promote the growth and invasion of tumor cells. They could also participate in tumor angiogenesis, infiltrate into tumor microenvironment, and further protect tumor cells from being attacked by immune system 19. In our study, we also observed that high platelet count was one of the independent predictors of poor prognosis. Therefore, platelets might play a vital role in the prognosis of EJA patients.

An increase in serum ALP usually indicates the existence of bile obstruction and the bone metastasis of cancers. In some published studies, ALP has been reported to correspond to the prognosis. For example, Namikawa et al. 20 observed that high serum levels of ALP were one of the poor prognosis predictive factors in advanced gastric cancer patients who received chemotherapy. Recently, researchers from London Royal Marsden Hospital have constructed a prognostic model containing serum ALP in advanced or metastatic esophagogastric cancer patients, and this result was also validated by other two randomized controlled trials 21-23. Researchers from the Yale Cancer Center also built another novel model for advanced gastric cancer patients using twelve indicators, among which ALP, LY, and NE were all inflammation-related markers 24. In our study, we observed that serum ALP was also concluded, and patients with high ALP preferred to the poor prognosis, which was similar to the results of the above-referred publications 21, 24.

It is widely accepted that cancer can stimulate the immune system to secrete antibodies against tumors. Some publications have suggested that the detection of serum antibodies could help identify cancers and predict prognosis. Ishdorj et al. 25 observed that in chronic lymphoblastic leukemia, low serum levels of IgG and high serum levels of IgA were two vital prognostic markers, indicating the worse prognosis. Along with the antigen-antibody reaction system, complements were also found to play a vital role in the body immune response. Research showed that the overexpression of complement C3 can strengthen the malignant progress of gastric cancer by activating the JAK2/STAT3 signaling pathway, and finally lead to a poor 5-year OS 26. Moreover, other studies have found that plasma levels of CFB in pancreatic cancer patients were two-fold higher than those of normal volunteers. They also found that the diagnostic efficiency of CFB was better than carbohydrate antigen 199 (CA199), and patients with high CFB levels had worse prognosis 27, 28. In our present study of EJA patients, preoperative high IgA, high C3, high CFB, and low IgG are all poor prognosis-related factors, which might be consistent with other cancer research mentioned above. Further study is needed to explore the related mechanisms in EJA.

SII is another inflammation-related indicator in our novel constructed nomogram, and the mathematics combination of three indicators, including blood neutrophil, lymphocyte, and platelet. It was first proposed when discussing the indicators in prognosis prediction of hepatocellular carcinoma patients 29. In this study, we observed that a high preoperative SII might be a protective factor in EJA patients' prognosis, which was inconsistent with the result of Jomrich et al 6. The difference might result from the different study subjects, as the latter contained patients receiving neoadjuvant chemoradiotherapy. However, our study enrolled a larger sample size than Jomrich et al. Therefore, the real predictive ability of SII should be further validated in the future.

Some innovations of this study could be listed as follows. First, some indicators we finally included into the nomogram were first reported, such as IgA, IgG, C3, and so on. Second, our nomogram in this study was novel, that was to say, no report has been carried out with the combination of the same twelve indicators so far. Third, the published nomograms by other researchers were mostly aimed at advanced EJA patients who only received chemoradiotherapy 21, 24. Indeed, two studies have constructed nomograms using other indicators for EJA patients receiving curative surgery resection with the C-indexes of 0.76 and 0.69 11, 30, which were consistent with our results. Importantly, our results could be more solid as we analyzed in a large sample size with validation. Fourth, most studies revealed prognostic predictive nomograms based on the Surveillance, Epidemiology, and End Results database 31-33. In our current study, we collected, analyzed, and built the nomogram based on our individual data with independent validation. Finally, as far as we know, we included the largest number of parameters (i.e. 50 preoperative clinical laboratory test results and sociodemographic characteristics) to build a nomogram for EJA prognosis to date.

However, some nonignorable limitations should not be ignored in this retrospective study. First, as a single-center study, our nomogram should be further validated in other institutes. Second, the follow-up of a part of patients in the validation group is below five years. In the future, we will continue to follow up these patients to further evaluate this novel nomogram. Third, as shown in Table 1, patients in the validation group were older than those in the training group, which might result in some bias. Nevertheless, although the age difference existed, the nomogram still showed robust performance and calibration ability in the validation group. In the published studies, we found that the researchers also included it into the final models although the age difference also existed 34-39. Based on these studies, we thought that it was not necessary to exclude indicators of which the distribution difference was significant between training and validation groups. Finally, there were no specific tumor indicators used for the diagnosis or prognosis prediction of EJA in clinical practice, although some studies have found that traditional cancer biomarkers including CA125 and CA199 might be used as prognostic indicators for EJA 30, 40. What's more, in this retrospective study, not all patients received the test of tumor indicators, which limited the application of these tumor markers for the construction of our nomogram. Whether the combination of tumor markers and our current nomogram would get a better performance or not should be further studied.

## Conclusions

To sum up, we evaluated fifty markers mainly consisting of preoperative clinical laboratory blood results and built a nomogram based on twelve indicators to predict the prognosis of curatively resected EJA patients. Most of the including indicators are inflammation- or immune-related indicators. In the future, if the further evaluation was carried out, this novel nomogram might provide a novel direction for the individual treatment options of EJA patients.

## Supplementary Material

Supplementary table.

## Figures and Tables

**Table 1:** Characteristics of EJA patients in the training and validation groups.

	Training group (n=465)	Validation group (n=289)	*p*
**Median OS (months)**	39.2 ± 5.9	38.0 ± 6.6	0.944
**Age, mean ± SD, years**	61.0 ± 8.1	64.0 ± 7.3	<0.001
**Gender**			0.099
Male	389	228	
Female	76	61	
**BMI, mean ± SD, kg/m^2^**	21.2 ± 2.8	21.3 ± 2.5	0.836
**Tumor family**			0.958
No	400	249	
Yes	65	40	
**Smoking history**			0.659
No	165	98	
Yes	300	191	
**Drinking history**			0.955
No	350	217	
Yes	115	72	
**TNM stage**			0.237
I (IA+IB+IC)	36	20	
II (IIA+IIB)	112	58	
III (IIIA+IIIB)	238	170	
IV (IIIC+IVA)	79	41	
**Siewert's classification**			0.694
Siewert's type I/II	270	172	
Siewert's type III	195	117	

Note: EJA: esophagogastric junction adenocarcinoma; OS: overall survival; SD: standard deviation; BMI: body mass index.

**Table 2:** The C-index of OS nomogram for predicting OS of EJA patients.

	C-index	95% CI	*p*
**Training group**			
TNM system	0.62	0.59 ~ 0.65	Ref.
OS nomogram	0.65	0.62 ~ 0.68	0.044
OS nomogram + TNM system	0.71	0.68 ~ 0.74	<0.001
**Validation group**			
TNM system	0.62	0.58 ~ 0.65	Ref.
OS nomogram	0.66	0.63 ~ 0.71	0.006
OS nomogram + TNM system	0.70	0.66 ~ 0.74	<0.001

Note: OS: overall survival; EJA: esophagogastric junction adenocarcinoma; CI: confidence interval

## Abbreviations

EJA: esophagogastric junction adenocarcinoma
OS: overall survival
C-index: Harrell's consistent index
NRI: net reclassification improvement
IDI: integrated discrimination improvement
BMI: body mass index
PLT: platelets
ALT: alanine transaminase
AST: aspartate aminotransferase
ALP: alkaline phosphatase
ALB: albumin
UA: uric acid
C3: complement C3
CFB: complement factor B
SII: systemic immune-inflammation index

## References

[1] Sung H, Ferlay J, Siegel RL, Laversanne M, Soerjomataram I, Jemal A (2021). Global Cancer Statistics 2020: GLOBOCAN Estimates of Incidence and Mortality Worldwide for 36 Cancers in 185 Countries. CA Cancer J Clin.

[2] Siewert JR, Stein HJ (1998). Classification of adenocarcinoma of the oesophagogastric junction. Br J Surg.

[3] Arnold M, Ferlay J, van Berge Henegouwen MI, Soerjomataram I (2020). Global burden of oesophageal and gastric cancer by histology and subsite in 2018. Gut.

[4] Haga Y, Hato S, Ikenaga M, Yamamoto K, Tsuburaya A, Doi K (2018). Validation of an assessment tool: Estimation of Postoperative Overall Survival for Gastric Cancer. Eur J Surg Oncol.

[5] Kudou K, Nakashima Y, Haruta Y, Nambara S, Tsuda Y, Kusumoto E (2021). Comparison of Inflammation-Based Prognostic Scores Associated with the Prognostic Impact of Adenocarcinoma of Esophagogastric Junction and Upper Gastric Cancer. Ann Surg Oncol.

[6] Jomrich G, Paireder M, Kristo I, Baierl A, Ilhan-Mutlu A, Preusser M (2021). High Systemic Immune-Inflammation Index is an Adverse Prognostic Factor for Patients With Gastroesophageal Adenocarcinoma. Ann Surg.

[7] Kudou K, Saeki H, Nakashima Y, Kamori T, Kawazoe T, Haruta Y (2019). C-reactive protein/albumin ratio is a poor prognostic factor of esophagogastric junction and upper gastric cancer. J Gastroenterol Hepatol.

[8] Rouzier R, Pusztai L, Garbay JR, Delaloge S, Hunt KK, Hortobagyi GN (2006). Development and validation of nomograms for predicting residual tumor size and the probability of successful conservative surgery with neoadjuvant chemotherapy for breast cancer. Cancer.

[9] Wang S, Yang L, Ci B, Maclean M, Gerber DE, Xiao G (2018). Development and Validation of a Nomogram Prognostic Model for SCLC Patients. J Thorac Oncol.

[10] Wen J, Yang Y, Liu P, Ye F, Tang H, Huang X (2017). Development and validation of a nomogram for predicting survival on the base of modified lymph node ratio in breast cancer patients. Breast.

[11] Wei ZJ, Qiao YT, Zhou BC, Rankine AN, Zhang LX, Su YZ (2022). Model established based on blood markers predicts overall survival in patients after radical resection of types II and III adenocarcinoma of the esophagogastric junction. World J Gastrointest Surg.

[12] Liu CT, Hong CQ, Huang XC, Li EM, Xu YW, Peng YH (2020). Blood-based Markers in the Prognostic Prediction of Esophagogastric Junction Cancer. Journal of Cancer.

[14] Chen L, Qian J, Lin L, Lin J, Chen Q, Zhuang Z (2021). Prognostic value of preoperative lymphocyte-to-monocyte ratio in oral cancer patients and establishment of a dynamic nomogram. Oral Dis.

[15] He Y, Mao M, Shi W, He Z, Zhang L, Wang X (2019). Development and validation of a prognostic nomogram in gastric cancer with hepatitis B virus infection. J Transl Med.

[16] Horak ER, Leek R, Klenk N, LeJeune S, Smith K, Stuart N (1992). Angiogenesis, assessed by platelet/endothelial cell adhesion molecule antibodies, as indicator of node metastases and survival in breast cancer. Lancet.

[17] Reijnen C, IntHout J, Massuger L, Strobbe F, Kusters-Vandevelde HVN, Haldorsen IS (2019). Diagnostic Accuracy of Clinical Biomarkers for Preoperative Prediction of Lymph Node Metastasis in Endometrial Carcinoma: A Systematic Review and Meta-Analysis. Oncologist.

[18] Catal O, Ozer B, Sit M (2020). Prediction of Lymph Node Metastasis in Colon Cancer via Platelet to Lymphocyte Ratio and Platelet Count. J Coll Physicians Surg Pak.

[19] Haemmerle M, Stone RL, Menter DG, Afshar-Kharghan V, Sood AK (2018). The Platelet Lifeline to Cancer: Challenges and Opportunities. Cancer Cell.

[20] Namikawa T, Ishida N, Tsuda S, Fujisawa K, Munekage E, Iwabu J (2019). Prognostic significance of serum alkaline phosphatase and lactate dehydrogenase levels in patients with unresectable advanced gastric cancer. Gastric cancer: official journal of the International Gastric Cancer Association and the Japanese Gastric Cancer Association.

[21] Chau I, Norman AR, Cunningham D, Waters JS, Oates J, Ross PJ (2004). Multivariate prognostic factor analysis in locally advanced and metastatic esophago-gastric cancer-pooled analysis from three multicenter, randomized, controlled trials using individual patient data. J Clin Oncol.

[22] Koo DH, Ryoo BY, Kim HJ, Ryu MH, Lee SS, Moon JH (2011). A prognostic model in patients who receive chemotherapy for metastatic or recurrent gastric cancer: validation and comparison with previous models. Cancer Chemother Pharmacol.

[23] Takahari D, Boku N, Mizusawa J, Takashima A, Yamada Y, Yoshino T (2014). Determination of prognostic factors in Japanese patients with advanced gastric cancer using the data from a randomized controlled trial, Japan clinical oncology group 9912. Oncologist.

[24] Fuchs CS, Muro K, Tomasek J, Van Cutsem E, Cho JY, Oh SC (2017). Prognostic Factor Analysis of Overall Survival in Gastric Cancer from Two Phase III Studies of Second-line Ramucirumab (REGARD and RAINBOW) Using Pooled Patient Data. J Gastric Cancer.

[25] Ishdorj G, Streu E, Lambert P, Dhaliwal HS, Mahmud SM, Gibson SB (2019). IgA levels at diagnosis predict for infections, time to treatment, and survival in chronic lymphocytic leukemia. Blood Adv.

[26] Yuan K, Ye J, Liu Z, Ren Y, He W, Xu J (2020). Complement C3 overexpression activates JAK2/STAT3 pathway and correlates with gastric cancer progression. J Exp Clin Cancer Res.

[27] Kim SH, Lee MJ, Hwang HK, Lee SH, Kim H, Paik YK (2019). Prognostic potential of the preoperative plasma complement factor B in resected pancreatic cancer: A pilot study. Cancer biomarkers: section A of Disease markers.

[28] Lee MJ, Na K, Jeong SK, Lim JS, Kim SA, Lee MJ (2014). Identification of human complement factor B as a novel biomarker candidate for pancreatic ductal adenocarcinoma. J Proteome Res.

[29] Hu B, Yang XR, Xu Y, Sun YF, Sun C, Guo W (2014). Systemic immune-inflammation index predicts prognosis of patients after curative resection for hepatocellular carcinoma. Clin Cancer Res.

[30] Chen J, Xia YJ, Liu TY, Lai YH, Yu JS, Zhang TH (2021). Development and validation of a survival nomogram for patients with Siewert type II/III adenocarcinoma of the esophagogastric junction based on real-world data. BMC cancer.

[31] Guo Z, Wang N, Liu F, Zhao Q (2022). Prognostic nomogram for Siewert type II adenocarcinoma of the esophagogastric junction patients with and without neoadjuvant radiotherapy: a retrospective cohort study. American journal of translational research.

[32] Liu X, Jiang Q, Yue C, Wang Q (2021). Clinicopathological Characteristics and Survival Predictions for Adenocarcinoma of the Esophagogastric Junction: A SEER Population-Based Retrospective Study. Int J Gen Med.

[33] Chen K, Deng X, Yang Z, Yu D, Zhang X, Zhang J (2020). Survival nomogram for patients with metastatic siewert type II adenocarcinoma of the esophagogastric junction: a population-based study. Expert Rev Gastroenterol Hepatol.

[34] Mu X, Li Y, He L, Guan H, Wang J, Wei Z (2021). Prognostic nomogram for adenoid cystic carcinoma in different anatomic sites. Head Neck.

[35] Jia Z, Yan Y, Wang J, Yang H, Zhan H, Chen Q (2021). Development and validation of prognostic nomogram in ependymoma: A retrospective analysis of the SEER database. Cancer medicine.

[36] Hu MD, Chen SH, Liu Y, Jia LH (2019). Development and validation of a nomogram to predict the prognosis of patients with squamous cell carcinoma of the bladder. Bioscience reports.

[37] Zhanghuang C, Wang J, Yao Z, Li L, Xie Y, Tang H (2022). Development and Validation of a Nomogram to Predict Cancer-Specific Survival in Elderly Patients With Papillary Renal Cell Carcinoma. Front Public Health.

[38] Zheng YM, Chen J, Xu Q, Zhao WH, Wang XF, Yuan MG (2021). Development and validation of an MRI-based radiomics nomogram for distinguishing Warthin's tumour from pleomorphic adenomas of the parotid gland. Dentomaxillofac Radiol.

[39] Huang XT, Cai JP, Chen W, Huang CS, Li JH, Gan TT (2022). Establishment and validation of a nomogram for predicting overall survival of node-negative perihilar cholangiocarcinoma. Asian J Surg.

[40] Tokunaga R, Imamura Y, Nakamura K, Uchihara T, Ishimoto T, Nakagawa S (2015). Carbohydrate antigen 19-9 is a useful prognostic marker in esophagogastric junction adenocarcinoma. Cancer medicine.

