# Supplementary material for: A novel nomogram based on clinical blood indicators for prognosis prediction in curatively resected esophagogastric junction adenocarcinoma patients
Source: J Cancer. 2023 May 21;14(9):1553–61. doi: 10.7150/jca.83588 (PMC10266239; doi:10.7150/jca.83588)
Supplement: Supplementary file 1 — Supplementary table. [file jcav14p1553s1.pdf]

Supplementary Materials

Supplementary Table S1. The univariable and multivariable Cox regression analysis for OS of EJA patients

Variables	Univariable Cox analysis			Multivariable Cox analysis		
	**HR**	**95% CI**	***p***	**HR**	**95% CI**	***p***
Gender (male vs. female)	0.950	0.701 ~ 1.287	0.739			
Age (years; >65.00 vs. ≤65.00)	1.752	1.387 ~ 2.214	<0.001	1.497	1.177 ~ 1.904	0.001
BMI (kg/m^2; >19.72 vs. ≤19.72)	0.667	0.527 ~ 0.845	0.001	0.669	0.524 ~ 0.854	0.001
Tumor Family History (Y vs. N)	1.023	0.738 ~ 1.418	0.890			
Smoking History (Y vs. N)	0.979	0.772 ~ 1.241	0.862			
Drinking History (Y vs. N)	1.123	0.870 ~ 1.449	0.373			
WBC (*10^9/l; >6.42 vs. ≤6.42)	1.238	0.983 ~ 1.560	0.069			
RBC (*10^{12}/l; >4.85 vs. ≤4.85)	0.780	0.601 ~ 1.010	0.060			
HGB (g/l; >131.00 vs. ≤131.00)	0.766	0.611 ~ 0.961	0.021			
HCT (%; >39.00 vs. ≤39.00)	0.780	0.621 ~ 0.980	0.033			
MCV (fl; >94.30 vs. ≤94.30)	1.150	0.890 ~ 1.485	0.286			
MCH (pg; >28.50 vs. ≤28.50)	1.225	0.959 ~ 1.563	0.104			
MCHC (g/l; >326.00 vs. ≤326.00)	1.161	0.925 ~ 1.458	0.199			
PLT (*10^9/l; >243.00 vs. ≤243.00)	1.266	1.009 ~ 1.588	0.042	1.288	1.010 ~ 1.644	0.041
LY% (>41.00 vs. ≤41.00)	1.282	0.863 ~ 1.905	0.219			
LY (*10^9/l; >2.15 vs. ≤2.15)	1.135	0.890 ~ 1.449	0.308			
MO% (>5.40 vs. ≤5.40)	0.841	0.634 ~ 1.115	0.229			
MO (*10^9/l; >0.34 vs. ≤0.34)	0.849	0.636 ~ 1.133	0.266			
NE% (>68.70 vs. ≤68.70)	0.790	0.586 ~ 1.066	0.123			
NE (*10^9/l; >3.59 vs. ≤3.59)	1.250	0.983 ~ 1.589	0.069			
NLR (>1.17 vs. ≤1.17)	0.795	0.542 ~ 1.167	0.242			
PLR (>112.79 vs. ≤112.79)	0.916	0.727 ~ 1.155	0.459			
LMR (>6.05 vs. ≤6.05)	1.287	0.945 ~ 1.753	0.110			
CK (U/l; >75.30 vs. ≤75.30)	0.746	0.594 ~ 0.936	0.011			
LDH (U/l; >149.20 vs. ≤149.20)	0.815	0.649 ~ 1.023	0.078			
HBDH (U/l; >134.90 vs. ≤134.90)	0.789	0.599 ~ 1.040	0.093			
AST (U/l; >16.00 vs. ≤16.00)	0.786	0.586 ~ 1.053	0.107			
AST/ALT (>1.21 vs. ≤1.21)	1.362	1.057 ~ 1.755	0.017	1.317	1.012 ~ 1.714	0.041
ALP (U/l; >59.00 vs. ≤59.00)	1.614	1.182 ~ 2.205	0.003	1.579	1.149 ~ 2.169	0.005
GGT (U/l; >37.80 vs. ≤37.80)	0.739	0.483 ~ 1.130	0.163			
TP (g/l; >67.90 vs. ≤67.90)	0.818	0.652 ~ 1.028	0.084			
ALB (g/l; >43.90 vs. ≤43.90)	0.758	0.599 ~ 0.960	0.022	0.760	0.592 ~ 0.975	0.031
GLB (g/l; >26.70 vs. ≤26.70)	1.193	0.951 ~ 1.498	0.128			
AGR (>1.78 vs. ≤1.78)	0.728	0.556 ~ 0.953	0.021			
TBIL (umol/l; >13.20 vs. ≤13.20)	0.868	0.691 ~ 1.088	0.219			
DBIL (umol/l; >2.30 vs. ≤2.30)	1.287	0.970 ~ 1.709	0.080			
IBIL (umol/l; >11.40 vs. ≤11.40)	0.818	0.645 ~ 1.038	0.099			
Glu (mmol/l; >5.01 vs. ≤5.01)	0.809	0.643 ~ 1.017	0.070			
Cr (umol/l; >105.10 vs. ≤105.10)	0.841	0.658 ~ 1.075	0.167			
UA (umol/l; >372.90 vs. ≤372.90)	1.380	1.069 ~ 1.781	0.013	1.455	1.120 ~ 1.888	0.005

IgA (g/l; >1.82 vs. ≤1.82)	1.290	1.024 ~ 1.624	0.030	1.391	1.099 ~ 1.762	0.006
IgG (g/l; >15.14 vs. ≤15.14)	0.551	0.375 ~ 0.808	0.002	0.557	0.374 ~ 0.828	0.004
IgM (g/l; >0.87 vs. ≤0.87)	0.818	0.623 ~ 1.076	0.151			
C3 (g/l; >0.98 vs. ≤0.98)	1.283	1.019 ~ 1.616	0.034	1.323	1.042 ~ 1.680	0.022
CFB (g/l; >0.40 vs. ≤0.40)	1.384	1.037 ~ 1.846	0.027	1.454	1.073 ~ 1.970	0.016
CRP (mg/l; >3.58 vs. ≤3.58)	1.227	0.977 ~ 1.541	0.079			
PNI (>55.39 vs. ≤55.39)	0.814	0.625 ~ 1.061	0.128			
NLR/ALB (>0.03 vs. ≤0.03)	0.874	0.596 ~ 1.283	0.493			
CRP/ALB (>0.10 vs. ≤0.10)	1.404	1.102 ~ 1.789	0.006			
SII (>253.60 vs. ≤253.60)	0.678	0.490 ~ 0.937	0.019	0.565	0.399 ~ 0.788	0.005
